# Midpalatal suture bone repair after miniscrew-assisted rapid palatal expansion in adults

**DOI:** 10.1186/s40510-022-00431-6

**Published:** 2022-10-17

**Authors:** Rodrigo Naveda, Alexandre Magno dos Santos, María Pía Seminario, Felicia Miranda, Guilherme Janson, Daniela Garib

**Affiliations:** 1 Department of Orthodontics, Bauru Dental School, University of São Paulo, São Paulo, Brazil; 2 Belo Horizonte, Minas Gerais Brazil; 3 Department of Orthodontics, Bauru Dental School, and Hospital for Rehabilitation of Craniofacial Anomalies, University of São Paulo, São Paulo, Brazil

**Keywords:** Palatal expansion technique, Skeletal anchorage, Cone-beam computed tomography

## Abstract

**Background:**

Midpalatal suture (MPS) repair in growing patients after RPE has been previously reported. However, differences between young and adult patients for timing and pattern of MPS repair after rapid maxillary expansion are expected. The aim of this study was to evaluate the midpalatal suture repair pattern after miniscrew-assisted rapid palatal expansion (MARPE) in adult patients.

**Materials and methods:**

The study included 21 patients (six males, 15 females) successfully treated with MARPE with a mean initial age of 29.1 years of age (SD = 8.0; range = 20.1–45.1). MPS repair was evaluated using maxillary axial and coronal sections derived from CBCT exams taken 16 months after the expansion (SD = 5.9). Objective and subjective assessments of MPS repair were performed. Objective assessments were performed measuring MPS bone density at anterior, median and posterior region of hard palate. Pre-expansion and post-retention bone density changes were evaluated using paired *t* tests (*p* < 0.05). Midpalatal suture bone repair was scored 0 to 3 considering, respectively, the complete absence of bone repair in the MPS, the repair of less than 50% of the MPS, the repair of more than 50% of the MPS and the complete repair of the MPS. Intra- and interexaminer reliability evaluation were assessed using Kappa coefficient.

**Results:**

The objective evaluation showed a significant higher bone density at the pre-expansion stage in all palatal regions. The reliability of the subjective method was adequate with intra- and interexaminer agreements varying from 0.807 to 0.904. Scores 1, 2 and 3 were found in 19.05%, 38.09% and 42.86% of the sample, respectively. The most common region demonstrating absence of bone repair was the middle third. The anterior third of the midpalatal suture was repaired in all patients.

**Conclusions:**

A decreased bone density was observed after the retention period when compared to pre-expansion stage. Most adult patients demonstrated incomplete repair of the midpalatal suture 16 months after MARPE. However, adequate bone repair covering more than half of the hard palate extension was observed in 80.95% of the patients.

## Background

Midpalatal suture (MPS) split has proven to be an adequate method for treatment of maxillary constriction and moderate maxillary crowding [1–3]. Conventional rapid palatal expansion (RPE) has the increasing age as a limitation to achieve maxillary transverse separation. Recently, miniscrew-assisted rapid palatal expansion (MARPE) has widen the age limit for midpalatal suture split allowing treatment of maxillary constriction in mature patients [4].

Immediate skeletal and dental effects of MARPE in adult patients have been previously studied. A pyramidal expansion pattern with more dental effects, similar to conventional rapid palatal expansion, was observed [5]. Skeletal transverse dimensions at the level of the nasal cavity, maxillary basal bone and alveolar ridge increased significantly after MARPE [5–8]. The skeletal effect corresponded to approximately 43.84% of the amount of screw activation [5]. Molars, premolars and canines widths also increased significantly after expansion [5–8]. Skeletal effects showed good stability in the long term, with no significant relapse after orthodontic treatment [5–8]. Dental effects decreased significantly after comprehensive orthodontic treatment, however, with no relapse of posterior crossbite [5–8]. The question that rises is whether the midpalatal suture repair after MARPE in mature patients is similar to that observed in growing patients.

Midpalatal suture repair in growing patients after RPE has been previously reported [9, 10]. Melsen histologically evaluated MPS repair after RPE in children of 8 to 13 years of age [10]. Evidence of inflammation with intense osteoblastic activity was reported after the first month of retention. After 5 to 6 months, bone islands along the suture were observed, and after 1 year of retention, a complete repaired suture was observed [10]. Ekstrom radiographically evaluated the MPS repair in a 10-year-old boy treated with RPE, calculating the mineral mass per surface unit [9]. After 3 months of retention, the MPS showed well-established mineralization, similar to the initial level [9]. Tomographic evaluation performed in a sample of 17 children ranging from 5 to 10 years showed a completely ossified suture after 8 to 9 months of retention [11]. A bone scintigraphy study evaluated bone activity in one pre-adolescent and two teenager patients after RPE [12]. Greater bone activity in the anterior and medial sections was observed during the first 3 months of retention. After this period, bone activity returned to the original level [12]. MARPE has proven to be and effective treatment for adult patients [13, 14]. However, MPS repair after MARPE in adult patients was not previously described. Bone repair has been related to initial age and amount of bone separation [15]. Previous studies with surgically assisted rapid palatal expansion (SARPE) in adults showed absence of complete sutural repair after 3 to 7 months of retention [16–18].

Differences between young and adult patients for timing and pattern of MPS repair after rapid maxillary expansion are expected. Midpalatal suture repair after MARPE is important to be assessed in order to define an adequate protocol of post-expansion retention. Therefore, the objective of this study was to evaluate bone repair after midpalatal suture split with MARPE in adults and to propose a classification method of midpalatal suture repair.

## Materials and methods

This retrospective study was approved by the institutional Research Ethics Committee of Bauru Dental School, University of São Paulo (process #22084619.5.0000.5417). Sample size calculation was based on a standard deviation for median bone density of 125 [17], a minimum intragroup difference of 100 Hounsfield units (HU), an alpha value of 5%, and a statistical power of 80%. The sample size was 14 subjects.

The sample included 24 consecutive patients treated with MARPE at a private practice by one orthodontist and two postgraduation programs. The inclusion criteria were patients older than 20 years of age with unilateral or bilateral posterior crossbite, successful MARPE therapy with radiographic confirmation of midpalatal suture split, and cone-beam computed tomography (CBCT) taken at least 6 months after expansion, for bone repair assessment. The exclusion criteria were presence of craniofacial anomalies and syndromes. From the total sample, three patients were excluded due to age younger than 20 years. The final sample comprised 21 patients (6 male, 15 female) with a mean initial age of 29.1 years of age (SD = 8.0; range = 20.1–45.1).

**Fig. 1:**
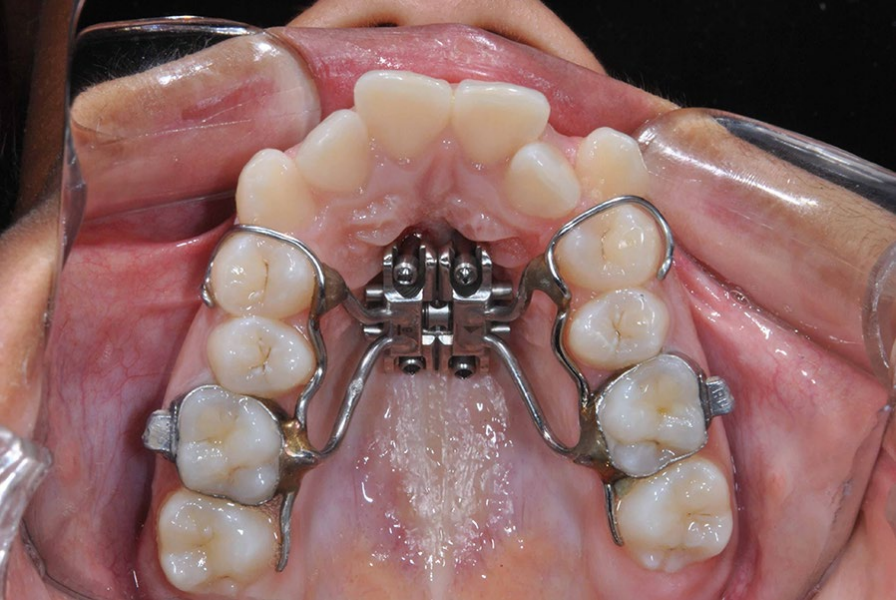
Expander used for MARPE

All expansion procedures were performed using a prefabricated expander (PecLab, Belo Horizonte, Brazil) shown in Fig. 1. The expander consisted of a MARPE expander with four paramedian miniscrews of 1.8 × 7 mm. The expander was positioned approximately in the middle third of the hard palate. The activation protocol initiated with two-quarter turns immediately after installation, followed by one-quarter turn (0.2 mm) twice a day in the consecutive days. When an interincisal diastema was opened, the screw was activated one-quarter turn a day until reaching overcorrection of the crossbite. The active expansion phase was approximately 21 to 30 days with a mean screw activation of 7 mm. The mean split at the level of the prosthion measured with a digital calypter in the occlusal radiograph was 4.66 mm (SD = 1.37). The expander device was maintained as retention for 12 months. Fixed orthodontic appliance was installed approximately 6 months after the active expansion phase in all the patients.

**Fig. 2:**
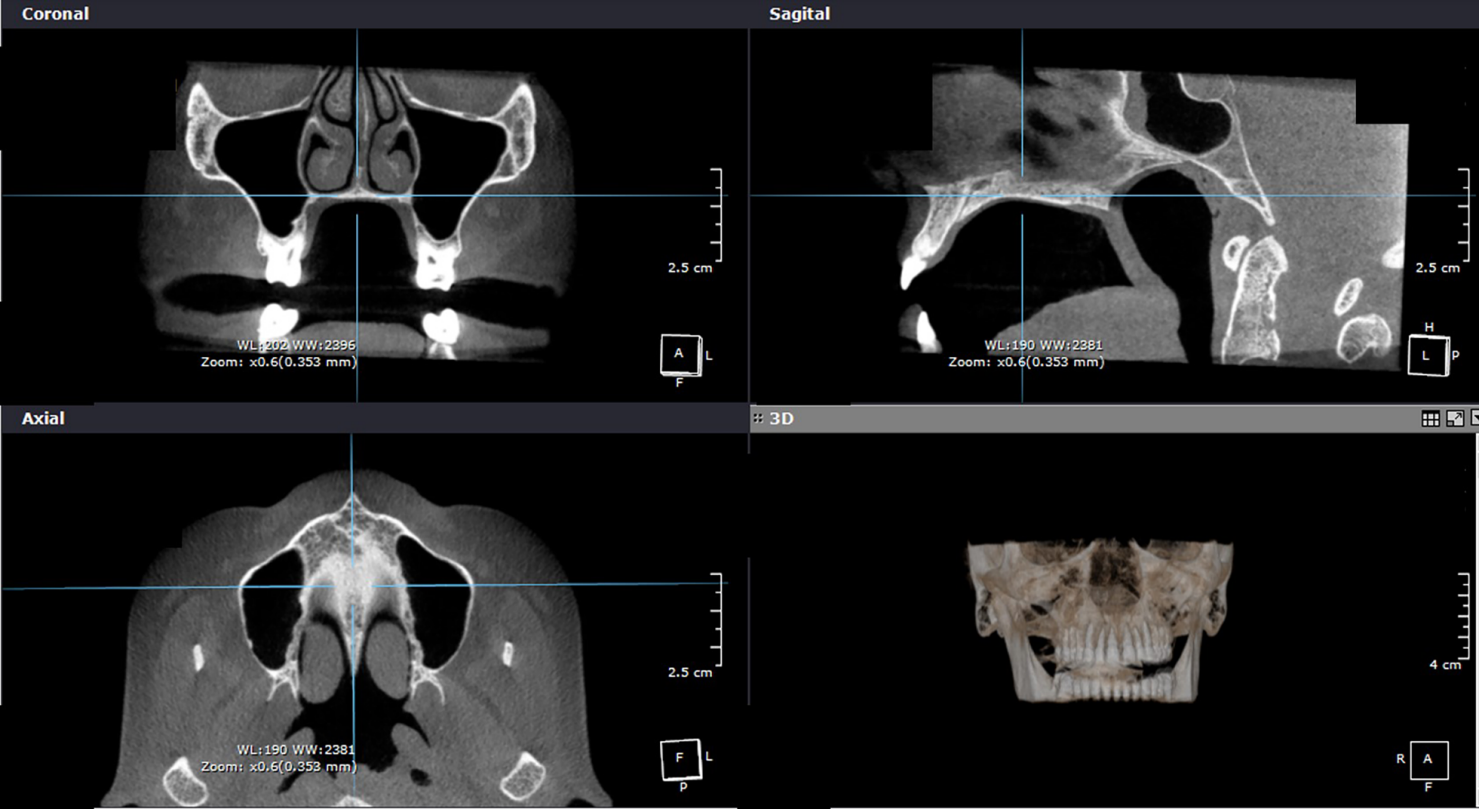
Standardization of image position

CBCT exams were obtained before expansion (T1) and after a retention period of at least 6 months (T2) using a FOV of 6 cm and a voxel size of 0.4 mm. The average time from the end of active expansion to the T2 CBCT exam was 16.5 ± 5.9 months. T1 and T2-CBCT derived axial sections of the hard palate were obtained. Image position standardization was performed in the three planes of space (Fig. 2). In the frontal view, the plane passing through the lower limit of the nasal cavity was left parallel to the horizontal plane. In the sagittal plane, a plane passing through the A point to the middle of posterior nasal spine was oriented parallel to the horizontal plane. In the axial view, the midpalatal suture was positioned perpendicular to the horizontal plane. Objective analysis was performed on the coronal sections by measuring bone density changes. Subjective analysis was performed in the axial sections by means of a qualitative visual analysis.

Bone density was measured before the expansion and after the retention period on CBCT coronal sections passing by the anterior, median and posterior regions of the hard palate. The anterior margin of the incisive foramen was reference for the anterior coronal section. The interproximal contact between maxillary right second premolar and first molar was the reference for the median coronal slice. The distal aspect of maxillary right second molar was the reference for the posterior coronal slice. An area of 2 × 2 mm was selected on the midpalatal suture at each coronal slice to determine the mean bone density using HU.

**Fig. 3:**
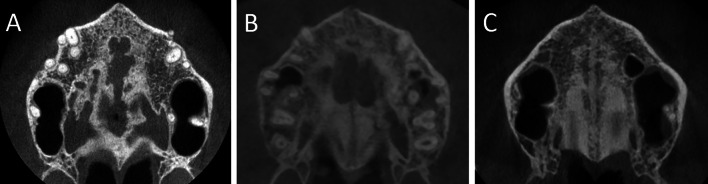
Examples of scores 1 to 3 for midpalatal bone repair after MARPE. **A** Score 1: incomplete bone repair in the midpalatal suture covering less than 50% of the hard palate; **B** Score 2: incomplete bone formation in the midpalatal suture with more than 50% of the hard palate demonstrating bone repair; **C** Score 3: complete repair of the midpalatal region extending from the anterior to the posterior region of the hard palate

In the T2 axial section, the degree of midpalatal bone repair was subjectively evaluated based on the presence/absence of visual bone at the MPS, and a score from 0 to 3 was assigned (Fig. 3). Score 0 represented the complete absence of bone repair in the MPS. Score 1 represented bone repair of less than 50% of the hard palate sagittal length (Fig. 3A). Score 2 demonstrated bone repair of more than 50% of the midpalatal suture (Fig. 3B). Score 3 was observation of complete repair of the midpalatal suture from the anterior to the posterior limit of the hard palate (Fig. 3C). The pre- and post-retention axial images of all patients were organized in a presentation as shown in Fig. 3 (Microsoft Office PowerPoint 2019; Microsoft, Redmont, Wash).

In order to evaluate reliability of the new evaluation method, the assessment was performed twice by three orthodontists. The three raters had previous training using six subjects from all scores, and disagreements were openly discussed. After the preliminary training, the axial images of the 21 patients were presented to the three examiners in a Power Point presentation with black background in the same room and using the same high-definition monitor. After a 30-day interval, all the sample was scored again using a second presentation with different arrangement of the images. In both times, orthodontists blindly classified the images with the same room conditions.

### Statistical analysis

Variables showed normal distribution and paired *t* tests were used to evaluate T1–T2 changes in bone density at the midpalatal suture. Kappa coefficient was used to evaluate intra- and interexaminer reliability of subjective assessments. Frequencies were used to describe the sample distribution among each bone repair score.

## Results

Objective evaluation showed a significant decrease in bone density from pre-expansion to post-retention phase (Table 1). A bone density decrease of 33%, 77% and 52% in the anterior, median and posterior regions, respectively, was observed after the retention period.

**Table 1:** Measurement of midpalatal suture bone density at T1 and T2

Density (HU)	T1 (Pre-expansion)				T2 (Post-expansion)				T2–T1		*p*
	Mean	SD	Minimum	Maximum	Mean	SD	Minimum	Maximum	Absolute	Relative	
Anterior	841.22	320.94	353.42	1543.19	556.87	308.75	− 13.50	1247.93	− 284.35	− 33.80%	0.005*
Median	759.34	242.88	422.76	1287.42	172.38	229.48	− 119.17	678.40	− 586.96	− 77.29%	< 0.001*
Posterior	751.95	330.82	212.30	1511.37	359.93	424.07	− 319.96	1061.75	− 392.01	− 52.13%	0.008*

*Statistically significant at *p* < 0.05

Intra- and interexaminer reproducibility of subjective assessment showed substantial to almost perfect agreement, with kappa coefficients varying from 0.807 to 0.904 (Table 2).

**Table 2:** Regeneration stage intra- and interexaminer reproducibility

Intraexaminer error		Interexaminer error	
Examiners	Kappa coefficient	Examiners	Kappa coefficient
1–1	0.813	1–2	0.904
2–2	0.807	1–3	0.904
3–3	0.811	2–3	0.811

Subjective assessment of midpalatal suture bone repair demonstrated that no patients had score 0 (Table 3). Score 1 was the less frequent with 19.05% of the sample. Scores 2 and 3 were found in 38.09% and 42.86% of the sample, respectively.

**Table 3:** Distribution of the regeneration stages, initial age and time of retention

	Stage 1	Stage 2	Stage 3
# (%)	4 (19.05%)	8 (38.09%)	9 (42.86%)
Initial age (SD)	24.5 (5.0)	34.6 (7.1)	26.1 (7.4)
Retention time (SD)	17.7 (6.2)	18.4 (4.7)	14.2 (6.5)

Considering the hard palate anteroposterior dimension, the most common region demonstrating absence of bone repair was the middle third (Table 4). The subjective evaluation showed that the anterior third of the midpalatal suture was repaired in all patients.

**Table 4:** Distribution of the regenerated areas of the midpalatal suture

Palatal region	Anterior	Medium	Posterior
Frequency of bone repair	21 (100%)	9 (42.86%)	17 (80.85%)

## Discussion

This is the first study showing the degree and pattern of bone repair of the midpalatal suture after miniscrew-assisted rapid palatal expansion in adults. Previous studies that evaluated MPS repair in young patients after expansion showed complete repair after 9 to 12 months of retention [10, 11]. Even with the presence of cellular activity in the MPS, mature patients seem to present a lower degree of repair after RPE [12].

The bone density at the midpalatal suture decreased after expansion (Table 1). These results are in accordance with previous studies that evaluated the MPS repair in adults after SARPE [17, 18]. A study that evaluated the bone density of 16 patients treated with SARPE associated with bone-borne Dresden Distractor reported lower bone density values compared to preoperative levels [17]. Our results showed that the greater decrease in bone density occurred in the middle region of the palate followed by the posterior and anterior regions (Table 1). Conversely, previous studies evaluating bone repair after SARPE observed a greater decrease in bone density at the anterior region of the palate [17, 18]. These differences might be related to the injuries caused by the use of chisel in the anterior region of the palate in SARPE. Another study evaluated MPS repair in a sample of 14 patients with a mean age of 25.3 years successfully treated with SARPE [18]. The tomographic evaluation after 180 days of the expansion showed lower bone density than the initial values, suggesting that the retention period was not enough for bone mineralization in adults [18]. The evaluation of the occlusal radiographs of 21 patients also showed the absence of complete repair of the MPS after 120 days after SARPE [16].

Despite the absence of complete repair in 57.14% of the sample in the subjective assessment, 80.95% of the patients presented bone repair covering more than half of the hard palate, 16 months after expansion (Table 3). The absence of complete repair of the MPS after expansion in adult patients is not unusual, and the results of this study are in accordance with previous studies [16–21] suggesting a lower degree of regeneration when compared to younger patients.

In the present study, MPS showed a triangular opening shape with a greater split at the anterior nasal spine. Despite this opening pattern, subjective evaluation showed that the complete sample demonstrated bone repair in the anterior region of the hard palate. Seventeen out of 21 subjects presented the anterior and posterior third of the palate repaired (Table 4). On the other hand, the middle third of the hard palate was the most frequently unrepaired region, observed in 57.14% of the patients. These outcomes might be related to a greater vascular irrigation in the anterior and posterior regions of the hard palate [22]. The middle region of the hard palate shows less vascular irrigation [22]. Additionally, the fact that high forces of the expansion are located in the area surrounding the miniscrews [23] and that they are installed in the middle region of the palate could have negatively influenced bone repair at this area. These results are in accordance with a previous study with bone scintigraphy showing that the anterior region of the midpalatal suture often shows more bone activity after RPE [12].

Objective and subjective analysis were conducted in the present study. Both evaluations showed that a better MPS repair was obtained in the anterior region, followed by the posterior and median region of the palate, respectively (Tables 1, 4). Scores were used as an additional evaluation because the bone density measurement is limited to very small areas. Using a visual observation method allowed the evaluation of the entire suture extension. A previous study also used a qualitative method to evaluate MPS repair in growing patients [11]. Qualitative analyses are used routinely to evaluate bone graft success in cleft lip and palate (CLP) patients. The evaluation of clinical success of alveolar bone graft in subjects with CLP also take in consideration the visual of bone filling of the cleft extension [24–26]. On a clinical point of view, the proposed method is useful for clinical practice.

**Fig. 4:**
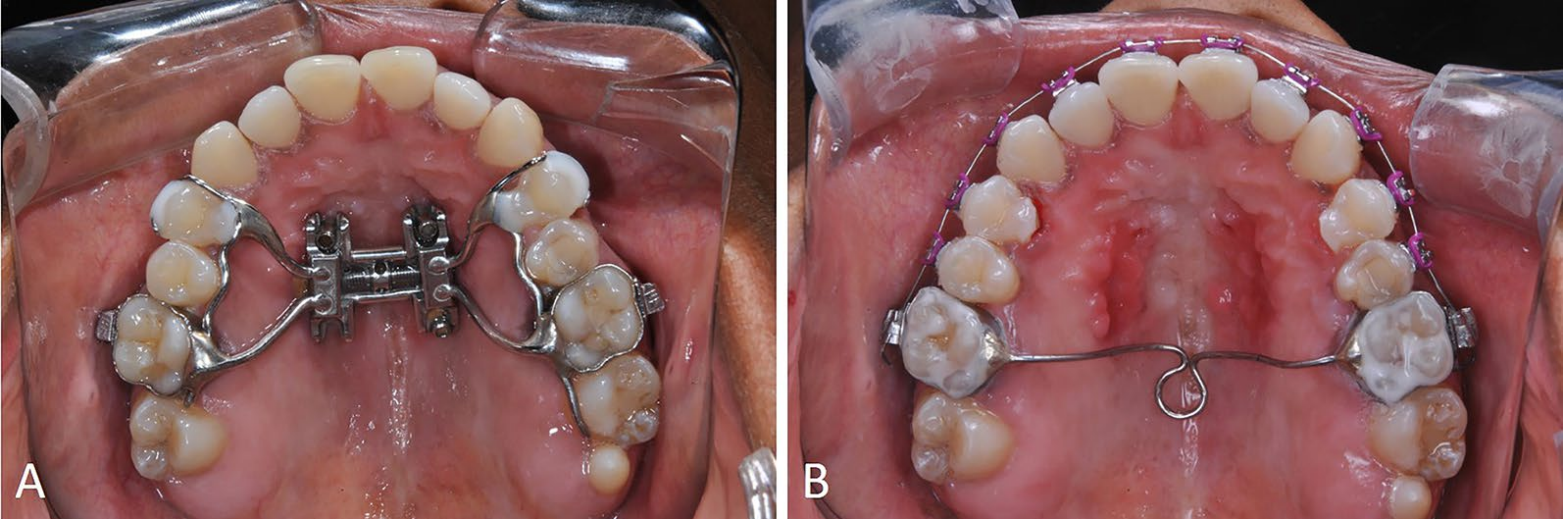
A transpalatal arch of 0.8mm stainless-steel wire installed immediately after expander removal

The absence of complete MPS repair observed in this study suggests that retention should be carefully planned after MARPE in order to maintain the transversal outcomes in adult patients. A transpalatal arch of 0.8-mm stainless steel wire should be installed immediately after expander removal (Fig. 4).

Despite the limitations of studying a sample with great initial age variance, the results of the present study provide preliminary information on MPS repair in adult patients after MARPE. Future studies should investigate the relationship of midpalatal suture repair and stability of the transverse results of MARPE. In addition, men and woman should be compared regarding the MPS repair after MARPE.

## Conclusions

- A decreased bone density was observed after the retention period when compared to pre-expansion stage;
- Most adult patients demonstrated incomplete repair of the midpalatal suture 16 months after MARPE;
- Bone repair covering more than half of the hard palate extension was observed in 80.95% of the patients;
- The middle third of the hard palate was the most frequently unrepaired region. Conversely, the anterior region of the hard palate showed bone formation in all patients after MARPE;
- The proposed scale for assessment of midpalatal suture bone repair after MARPE demonstrated adequate reliability.

## Data Availability

The raw data are present in the CBCT software of the orthodontic department of our university.

## Abbreviations

MPS: Midpalatal suture
RPE: Rapid palatal expansion
MARPE: Miniscrew-assisted rapid palatal expansion
SARPE: Surgically assisted rapid palatal expansion
CBCT: Cone-beam computed tomography
CLP: Cleft lip and palate
